# An *in vitro* model of drug-resistant seizures for selecting clinically effective antiseizure medications in Febrile Infection-Related Epilepsy Syndrome

**DOI:** 10.3389/fneur.2023.1129138

**Published:** 2023-03-22

**Authors:** Milica Cerovic, Martina Di Nunzio, Ilaria Craparotta, Annamaria Vezzani

**Affiliations:** ^1^Department of Neuroscience, Istituto di Ricerche Farmacologiche Mario Negri IRCCS, Milan, Italy; ^2^Department of Acute Brain Injury, Istituto di Ricerche Farmacologiche Mario Negri IRCCS, Milan, Italy; ^3^Department of Oncology, Istituto di Ricerche Farmacologiche Mario Negri IRCCS, Milan, Italy

**Keywords:** drug-refractory status epilepticus, neuroinflammation, antiseizure medications, immunomodulatory drugs, cytokines

## Abstract

**Introduction:**

FIRES is a rare epileptic encephalopathy induced by acute unremitting seizures that occur suddenly in healthy children or young adults after a febrile illness in the preceding 2 weeks. This condition results in high mortality, neurological disability, and drug-resistant epilepsy. The development of new therapeutics is hampered by the lack of validated experimental models. Our goal was to address this unmet need by providing a simple tool for rapid throughput screening of new therapies that target pathological inflammatory mechanisms in FIRES. The model was not intended to mimic the etiopathogenesis of FIRES which is still unknown, but to reproduce salient features of its clinical presentation such as the age, the cytokine storm and the refractoriness of epileptic activity to antiseizure medications (ASMs).

**Methods:**

We refined an *in vitro* model of mouse hippocampal/temporal cortex acute slices where drug-resistant epileptic activity is induced by zero Mg^2+^/100 μM 4-aminopirydine. Clinical evidence suggests that acute unremitting seizures in FIRES are promoted by neuroinflammation triggered in the brain by the preceding infection. We mimicked this inflammatory component by exposing slices for 30 min to 10 μg/ml lipopolysaccharide (LPS).

**Results:**

LPS induced a sustained neuroinflammatory response, as shown by increased mRNA levels of IL-1β, CXCL1 (IL-8), TNF, and increased IL-1β/IL-1Ra ratio. Epileptiform activity was exacerbated by neuroinflammation, also displaying increased resistance to maximal therapeutic concentrations of midazolam (100 μM), phenytoin (50 μM), sodium valproate (800 μM), and phenobarbital (100 μM). Treatment of LPS-exposed slices with two immunomodulatory drugs, a mouse anti-IL-6 receptor antibody (100 μM) corresponding to tocilizumab in humans, or anakinra (1.3 μM) which blocks the IL-1 receptor type 1, delayed the onset of epileptiform events and strongly reduced the ASM-resistant epileptiform activity evoked by neuroinflammation. These drugs were shown to reduce ASM-refractory seizures in FIRES patients.

**Discussion:**

The neuroinflammatory component and the pharmacological responsiveness of epileptiform events provide a proof-of-concept validation of this *in vitro* model for the rapid selection of new treatments for acute ASM-refractory seizures in FIRES.

## 1. Introduction

Febrile Infection-Related Epilepsy Syndrome (FIRES) is a “subcategory of New-Onset Refractory Status Epilepticus (NORSE)” that requires a prior febrile infection, with fever starting between 2 weeks and 24 h prior to onset of refractory status epilepticus (SE). NORSE is defined as a clinical presentation, not a specific diagnosis, in a patient without active epilepsy or other pre-existing relevant neurological disorder, and without a clear acute or active structural, toxic, or metabolic cause (1). After ineffectiveness of first and second line ASMs on acute seizures, at least 75% of FIRES patients require continuous infusion of anesthetics to stop seizures, with frequent relapses when anesthetics are discontinued (2–5).

FIRES etiopathogenesis remains unknown, however, both experimental and clinical research strongly suggest that neuroinflammation is a key precipitating factor. In particular, a febrile infection would trigger a self-perpetuating dysregulation of innate immunity involving glial cells and neurons in susceptible individuals (2). The neuroinflammatory response in FIRES patients is reflected by a storm of cytokines and chemokines, such as IL-1β, IL-6, TNF and IL-8 (6–9) in the CSF, as well as by reactive microglia and astrocytes (10) and increased expression of IL-1β and IL1 receptor type 1 (IL-1R1) in brain tissue (11). Activation of neuroinflammatory signallings in the brain promotes seizures in animal models (12, 13), thus suggesting that the cytokine storm contributes to the onset and perpetuation of seizures in FIRES.

Based on this evidence, new immunomodulatory/anti-inflammatory drugs in clinical use for other indications have been recently shown to reduce seizures in FIRES patients, such as systemic administration of anakinra or tocilizumab, or intrathecal dexamethasone (6, 14) [*reviewed in* (3, 15)]. In particular, these interventions were effective on unremitting seizures, also shortening the duration of mechanical ventilation, intensive care and hospital stay, and improving neurological outcomes (6, 14) [*reviewed in* (3, 15)].

There is a large arsenal of anti-inflammatory drugs to be repurposed for their therapeutic potential in NORSE/FIRES for stopping ASM-refractory seizures, thus preventing the long-term neurological consequences. There is urgent need, therefore, of developing experimental tools for rapid test of new drugs and for identifying molecular targets.

Since no validated experimental models for FIRES are as yet available, we set up a new *in vitro* model of drug-resistant seizures in FIRES. The model did not have to mimic the etiopathogenesis of FIRES, which is still unknown, but to provide a simple tool for rapid throughput screening of new therapies that target pathological inflammatory mechanisms in FIRES.

To this aim, we exposed hippocampal/temporal lobe slices from naïve mice of an age that approximates the clinical condition (16), to an inflammatory challenge. This challenge occurred before slices were exposed to hyperexcitable conditions evoking ASM-resistant seizures. Our data provide a proof-of-concept validation of this *in vitro* model for selecting treatments for the acute ASM-refractory seizures in FIRES.

## 2. Materials and methods

### 2.1. Animals and brain slice preparation

We used 28–30-day old male C57BL6/N mice. All procedures involving animals and their care were conducted in accordance with the principles set out in laws, regulations, and policies governing the care and use of laboratory animals: Italian Governing Law (D.lgs 26/2014; Authorisation n.19/2008-A issued March 6, 2008 by Ministry of Health); Mario Negri Institutional Regulations and Policies (Quality Management System Certificate—UNI EN ISO 9001:2008—Reg. N° 8576-A); the NIH Guide for the Care and Use of Laboratory Animals (2011 edition) and EU directives and guidelines (EEC Council Directive 2010/63/UE). Experiments were reviewed and approved by the intramural Animal Care and Use Committee, and by the Italian Ministry of Health.

Mice were killed by cervical dislocation. Brain was rapidly removed from the skull and horizontal brain slices (350 μm) from both hemispheres were cut with a vibratome (Leica VT 1000S) in ice-cold modified artificial cerebrospinal fluid (aCSF, mM): 87 NaCl, 2.5 KCl, 1 NaH_2_PO_4_, 75 sucrose, 7 MgCl_2_, 24 NaHCO_3_, 11 mM D-glucose, and 0.5 mM CaCl_2_. Then, slices were transferred into the incubating chamber and submerged in aCSF containing (mM): 130 NaCl, 3.5 KCl, 1.2 NaH_2_PO_4_, 1.3 MgCl_2_, 25 NaHCO_3_, 11 D-glucose, 2 CaCl_2_, and constantly bubbled with 95% O_2_ and 5% CO_2_ at room temperature. Slices were incubated in this condition for at least 1 h before starting the experiment.

### 2.2. High-density CMOS microelectrode array recordings

Recordings were performed using CMOS-microelectrode array (MEA) BioCamX (3Brain GmbH, Lanquart, Switzerland) at room temperature and slices were continuously perfused with oxygenated aCSF at a rate of 2 ml/min.

The recording array allowed simultaneous extracellular recordings from 4,096 electrodes at a sampling rate of 10 kHz per channel. The channels were arranged in a 64 × 64 array configuration and each square pixel measured 21 × 21 μm. The size of the recording area on the chip was suitable for recording from the entire hippocampal/cortical slice. Once the slice was positioned on the chip, it was held in place with a custom-made anchor of platinum wire and nylon mash.

The epileptiform activity was triggered by slice perfusion for 40 min with aCSF containing zero (0) Mg^2+^ and 100 μM 4-aminopyridine (4-AP) (17, 18), and consisted of synchronized field potentials (FPs) occurring at different frequency rates. Slices were preincubated for 30 min with aCSF alone or with the addition of 10 μg/ml lipopolysaccharide (LPS) before perfusion with the ictogenic cocktail. LPS was washed out for 5 min with aCSF before slice perfusion with 0 Mg^2+^ + 100 μM 4-AP (Supplementary Figure 1A). The tested drugs were added to the perfusion solution as shown in Supplementary Figures 1B, C (*see below*). Activity was recorded during 10 min sessions (T1 = 0–10 min; T2 = 20–30 min from the start of 0 Mg^2+^ + 100 μM 4-AP perfusion). FPs were detected using BrainWave5 software (3Brain) as follows: high and low threshold were set at +200 μV and −200 μV, energy window 40 ms, refractory period 5 ms and maximum wave duration 500 ms.

We classified the epileptiform activity evoked 0 Mg^2+^ + 100 μM 4-AP into three major categories: (1) *ictal events* consisting of synchronous and repetitive field potentials (FPs) discharges (>5 s) with frequency >1 Hz (*see tracing in* **Figure 3A**); (2) *status epilepticus* (SE)-like events consisting of repetitive FPs with frequency ranging between 0.8–1.3 Hz and lasting >5 min (*see* **Figure 3A**); (3) interictal events consisting of synchronous single or repetitive FPs (<5 s). FP bursts were identified by a minimum of three FPs/burst with an interval between FPs ≤1 s.

At the beginning of the experiment, a digital image of the slice was taken trough a stereomicroscope. During the *post-hoc* analysis of the epileptiform activities, the digital image was overlayed on the activity map to identify the active areas in the slice. For quantification of epileptiform activity (FP frequency, amplitude and burst duration; incidence of ictal and SE events), we focused the analysis on the most active area in the whole hippocampal/cortical slice, as determined by activity map and the corresponding raster plot (e.g., **Figures 2A**, **B**). FPs measures were reckoned by averaging the values from each electrode in the active area. *Post-hoc* analysis showed that the most active areas were randomly distributed among hippocampus and temporal cortex in the various experimental groups.

### 2.3. Drug application

Slices were incubated for 30 min in aCSF or LPS (+5 min wash-out), then 0 Mg^2+^ + 100 μM 4-AP perfusion was started. After recording recurrent epileptiform events for at least 10 min, slices were perfused with the selected ASMs (dissolved in the ictogenic cocktail) at their maximal therapeutic plasma concentration in humans (4) for 40 min: phenytoin 50 μM, phenobarbital 100 μM, sodium valproate 800 μM (Sigma-Aldrich, USA), midazolam 100 μM [Accord Helathcare Italia srl (19); *n* = 5–7 slices/drug from 3 to 5 mice]. The experimental protocol is described in Supplementary Figure 1B. For testing the immunomodulatory drugs, slices were pre-incubated with LPS in aCSF (30 min + 5 min washout), then perfused for 15 min with aCSF ± anakinra [22 μg/ml; 1.3 μM; Swedish Orphan Biovitrum AB (Sobi), Stockholm, Sweden] or chimeric mouse/rat anti-IL-6R antibody (14.7 μg/ml; 100 μM; Genetech Inc., San Francisco, CA, USA). Then, 0 Mg^2+^ + 100 μM 4-AP was perfused (± drugs) for 40 min (*n* = 6 slices /drug from 3 to 5 mice).

### 2.4. RTqPCR

We used an independent set of slices to determine the neuroinflammatory response to 10 μg/ml LPS. After 30 min LPS incubation, slices were washed out in aCSF for either 30 min or 60 min (*n* = 7–10 slices/time point). At the end of washout, slices were collected and snap-frozen in liquid nitrogen, then stored at −80°C until analysis. Tissue was homogenized in Qiazol Lysis Reagent (Qiagen, Hilden, Germania) and total RNA isolated using the miRNeasy Mini kit (Qiagen) according to manufacturer's instructions. The concentration and purity of RNA were determined at 260/280 nm using a high-speed microfluidic UV/VIS spectrophotometer QIAxpert (Qiagen) and the integrity and quality of RNA were evaluated by 4200 Tapestation (Agilent Technologies, Santa Clara, CA, USA). cDNA was synthesized from 800 ng RNA using the high-capacity cDNA reverse transcription kit (Applied Biosystems, Waltham, Massachusetts, USA) following the manufacturer's protocol (Applied Biosystems). RT-qPCR experiments were run in triplicate for each sample using 384-well reaction plates and an automatic liquid handling station (epMotion 5075LH, Eppendorf, Hamburg, Germany) on an Applied Biosystems 7900HT System (Applied Biosystems). mRNA expression was analyzed using QuantiFast SYBR Green PCR Master Mix (Qiagen). The designed primers are reported in Table 1. Data were normalized using geometric mean of 2 independent house-keeping genes (*s2Vb* and *Actb*). Cycle threshold (CT) values were obtained using manual threshold.

**Table 1:** Primer sequence.

**Gene**	**Primer sequence**	
	**Forward**	**Reverse**
*Actb*	GCCCTGAGGCTCTTTTCCAG	TGCCACAGGATTCCATACCC
*s2Vb*	AGCATGTCACTGGCCATCAA	CCCAATCCCTATGCCTGAGAT
*Il1rn*	AACCACCAGGGCATCACAT	CTTGCCGACATGGAATAAGG
*Il1b*	TGCCACCTTTTGACAGTGAT	GATGTGCTGCTGCGAGATT
*Tnf*	TGAACTTCGGGGTGATCG	GGTGGTTTGTGAGTGTGAGG
*Cxcl1*	ACCGAAGTGATAGCCACACTC	TCCGTTACTTGGGGACACC

### 2.5. Statistical analysis

Statistical analysis was performed by GraphPad Prism 8 (GraphPad Software, USA) for Windows using raw data. Data are presented as bargrams with individual values, and mean ± SEM. Non-parametric tests were chosen due to the low power of normality tests when the sample size is small. In each experiment, statistical analysis is reported in the respective figure legend. Differences between groups were considered significant for values of *p* < 0.05. Sample size was *a priori* determined based on literature data and previous experience with the 0 Mg^2^ +100 μM 4-AP model.

## 3. Results

### 3.1. Neuroinflammation *in vitro* model

We refined an *in vitro* model of epileptiform activities in hippocampal/temporal cortex slices from naive mice induced by 0 Mg^2+^ + 4-AP. The aim was to mimic the unresponsiveness of epileptiform events to ASMs (17), as observed in FIRES patients. We added the prototypical immune-inflammatory agent LPS to reproduce the cytokine storm that precedes seizure precipitation in FIRES.

**Figure 1:**
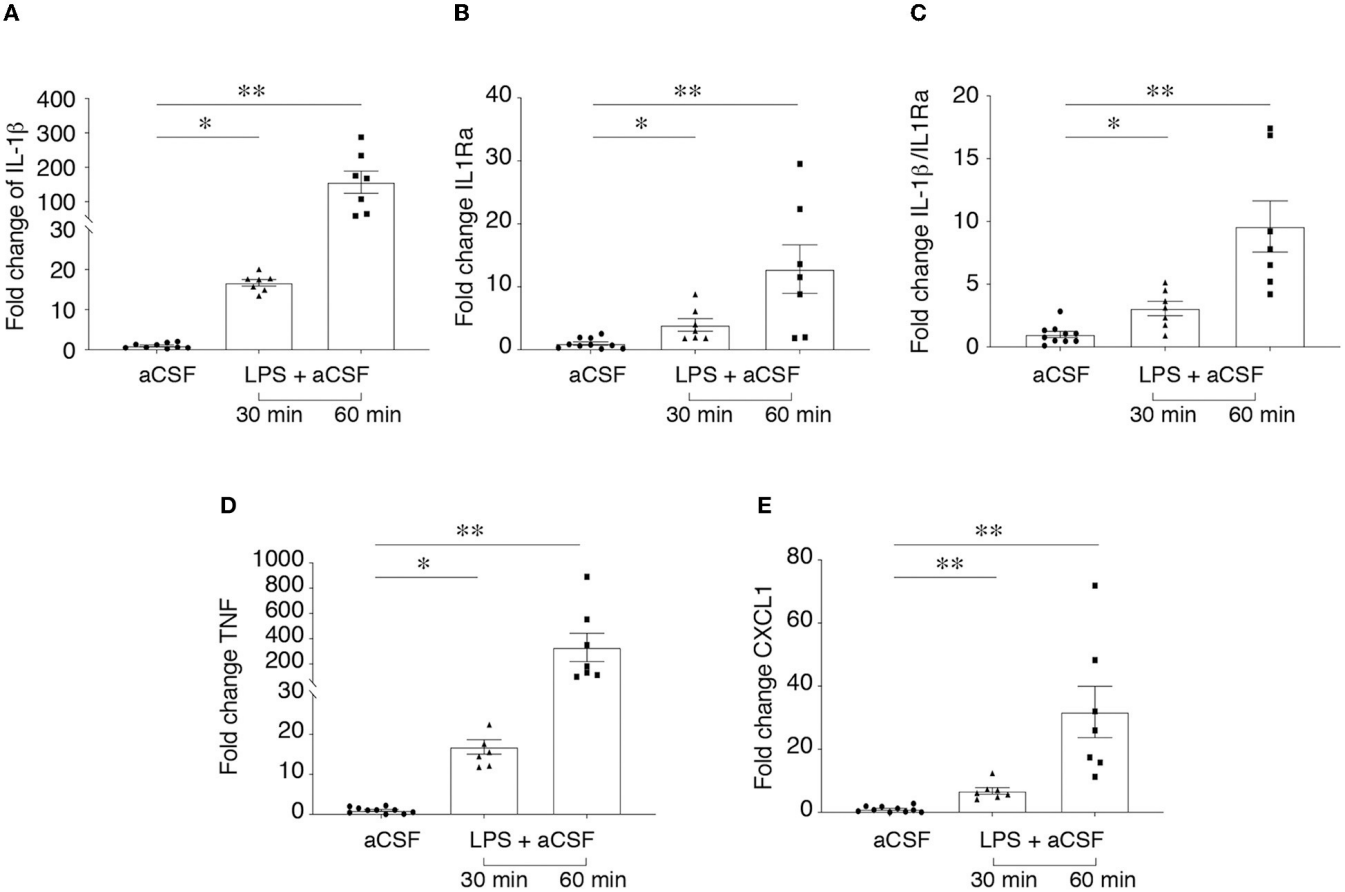
LPS-induced neuroinflammation in hippocampus/temporal cortex slices. RT-qPCR analysis of cytokine mRNA **(A–E)** in hippocampal/temporal cortex slices incubated with aCSF alone or with 10 μg/ml LPS for 30 min, followed by 30 min or 60 min washout in aCSF. Reference genes were *s2Vb and Actb*. Data are presented as fold-increase vs. control value in aCSF incubated slices (mean ± SEM and single values). *p < 0.05, **p < 0.01 vs. aCSF by Kruskal–Wallis test followed by Dunn's multiple comparison test.

Figure 1 shows a significant induction of ictogenic cytokines mRNA, namely *Il1b* **(A)**, *Tnf* **(D)** and *Cxcl1* **(E)** in brain slices after 30 min incubation with 10 μg/ml LPS. This induction was evident after 30 min LPS washout (*p* < 0.05; *p* < 0.01 vs. aCSF) and persisted after 60 min washout (*p* < 0.01 vs. aCSF). *Il1rn* (B) was also induced at both time points (*p* < 0.05; *p* < 0.01 vs. aCSF) although to a minor extent as compared to *Il1b* transcript. Accordingly, *Il1b*/*Il1rn* ratio **(C)** was significantly increased (*p* < 0.05; *p* < 0.01) compared to aCSF incubated slices, thus denoting a predominance of proinfammatory vs. antiinfammatory cytokines.

### 3.2. Exacerbation of epileptiform activity by neuroinflammation

**Figure 2:**
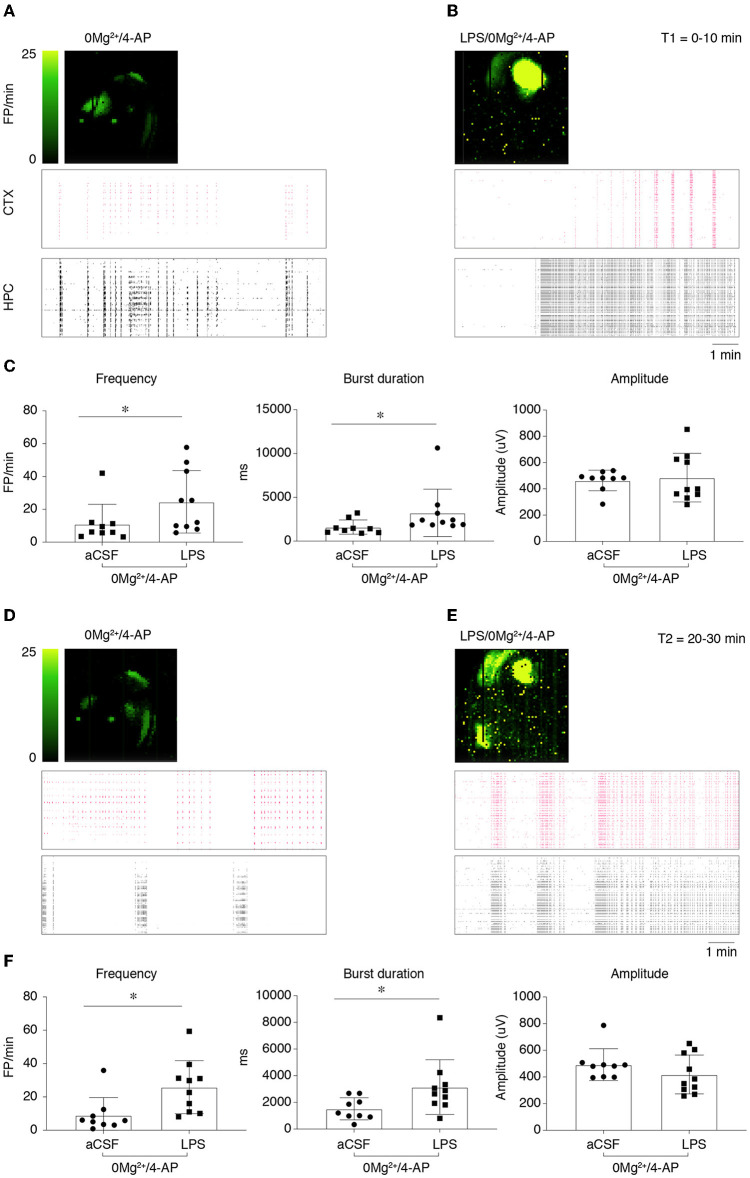
Lipopolysaccharide effect on epileptiform activity in hippocampus/temporal cortex slices. **(A, B)** Depict representative activity maps (*first row*; higher field potential, FP, frequency corresponds to lighter green color) and raster plots (*second and third rows*) of epileptiform activity recorded in the temporal cortex (CTX) or hippocampus (HPC) during 0 Mg^2+^/100 μM 4-AP perfusion alone **(A)** or in slices pre-incubated with LPS [**(B)**; 10 μg/ml LPS for 30 min, followed by 5 min washout], then exposed to 0 Mg^2+^/100 μM 4-AP for 40 min. **(C)** Reports quantification of epileptiform activity (FP frequency, burst duration and amplitude) *during T1* (0–10 min from the start of 0 Mg^2+^/4-AP perfusion) reckoned in the area of higher activity (as shown by activity map/raster plot) in each slice. **(D, E)** Depict representative activity maps and raster plots *during T2* (20–30 min from the start of 0 Mg^2+^/4-AP perfusion). **(F)** Reports quantification of epileptiform activity during T2 reckoned in the area of higher activity in each slice. Data are presented as mean ± SEM and single values (0 Mg^2+^ + 4-AP, *n* = 9 slices; LPS + 0 Mg^2+^ + 4AP, *n* = 10 slices). *p < 0.05 vs. 0 Mg^2+^/4AP by Mann–Whitney test.

**Figure 3:**
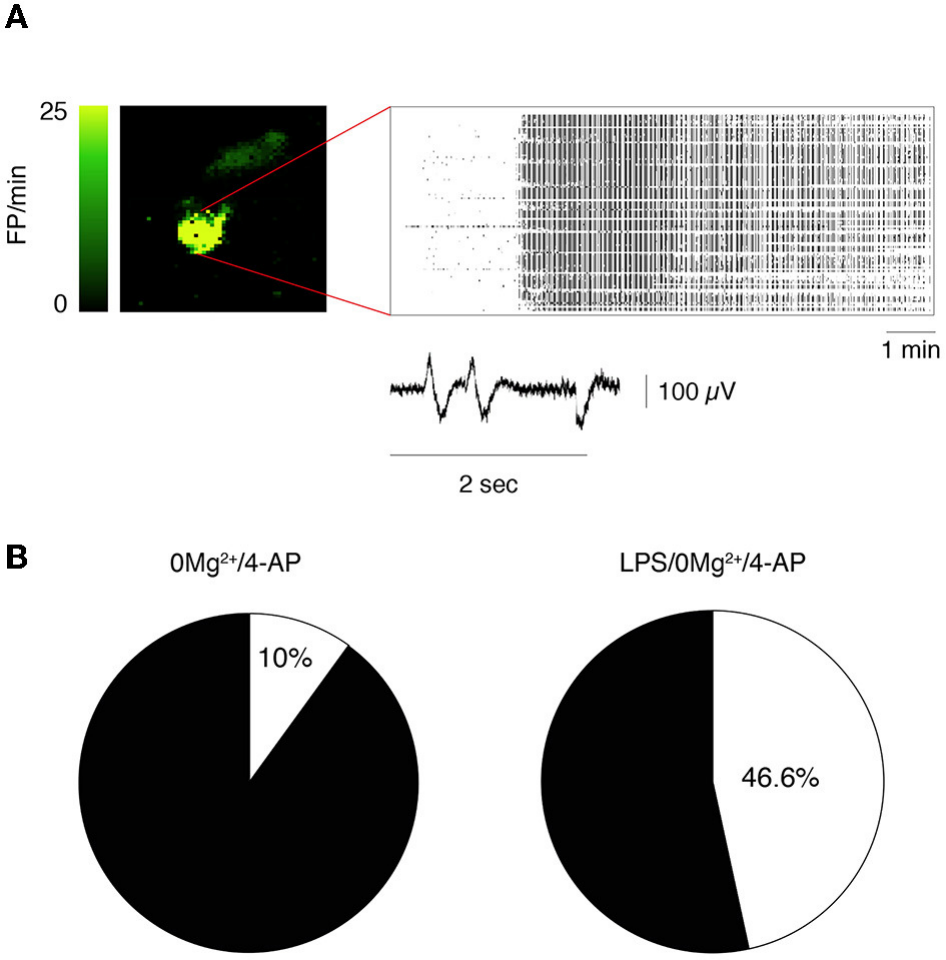
Incidence of status epilepticus in LPS-treated slices. **(A)** Depicts a representative activity map (higher FP frequency corresponds to lighter green color) and raster plot showing a status epilepticus (SE) event in hippocampus. Enlarged tracing depicts FPs from one representative electrode. **(B)** Shows the incidence of SE events in 0 Mg^2+^/4-AP exposed slices ± LPS (10 μg/ml LPS for 30 min, followed by 5 min washout). 0 Mg^2+^ + 4-AP, *n* = 9 slices; LPS+0 Mg^2+^ + 4AP, *n* = 10 slices. *p* < 0.05 vs. 0 Mg^2+^/4AP by Chi-square test.

Epileptiform activity (activity map and raster plot are depicted in Figures 2A, B, D, E) was quantified during 10 min recording at two sequential time points (T1= 0–10 min, Figures 2A, B and T2 = 20–30 min, Figures 2D, E) from the start of 0 Mg^2+^ + 4-AP perfusion. Pre-incubation for 30 min with LPS exacerbated epileptiform activity in slices (Figures 2B, E vs. Figures 2A, D): bargrams show an increased frequency of FPs (*p* < 0.05 vs. 0 Mg^2+^ + 4-AP alone) and FP burst duration (*p* < 0.05) at T1 (Figure 2C) and T2 (Figure 2F), without affecting the amplitude of recorded events. Moreover, the incidence SE events (Figure 3A; see Section 2.2 for definition) observed in 0 Mg^2+^ + 4-AP bathed slices was significantly increased by four-fold on average in LPS pre-incubated slices (Figure 3B; *p* < 0.05).

### 3.3. ASM-resistance of epileptiform activity is increased by neuroinflammation

**Figure 4:**
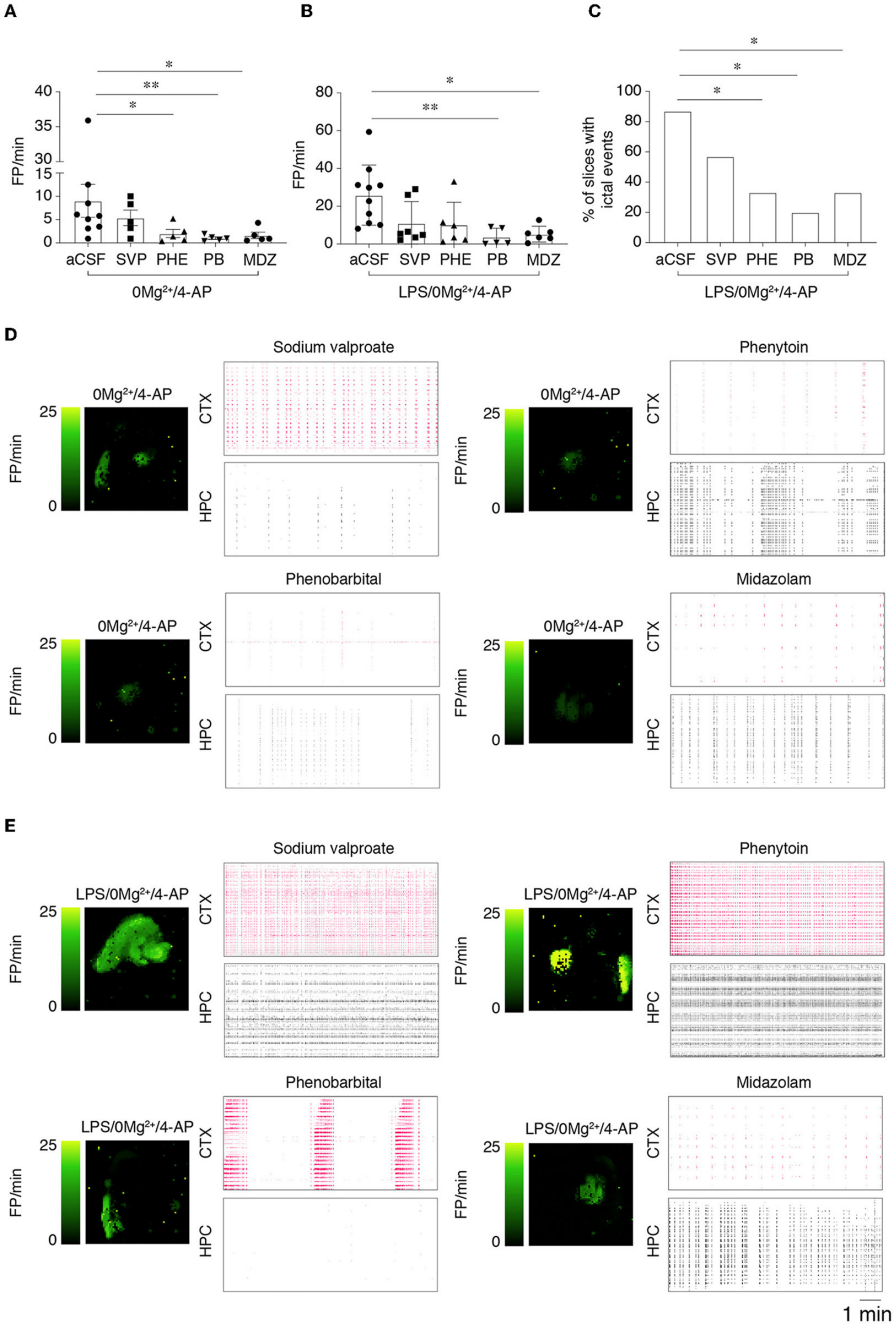
Effect of ASMs on epileptiform activities. **(A, B)** Show the effect of 800 μM sodium valproate (SVP), 50 μM phenytoin (PHE), 100 μM phenobarbital (PB) and midazolam (MDZ, 100 μM) on field potential (FP) frequency in slices perfused with 0 Mg^2+^/4-AP **(A)** or pre-treated with LPS **(B)** (10 μg/ml LPS for 30 min, followed by 5 min washout), then exposed to 0 Mg^2+^/100 μM 4-AP for 40 min. Quantification of epileptiform activity was done *during T2* (20–30 min from the start of 0 Mg^2+^/4-AP perfusion) in the area of higher activity in each slice. Data are presented as mean ± SEM and single values (*n* = 5–7 slices/experimental group) *p < 0.05; **p < 0.01 by Mann–Whitney test vs. respective control slices (aCSF, no ASMs added). **(C)** Shows the incidence of the combination of ictal and SE events in LPS-pretreated slices in the various experimental groups. *p < 0.05 vs. 0 Mg^2+^/4AP by Chi-square test. **(D, E)** Depict representative activity maps (higher FP frequency corresponds to lighter green color) and raster plots in temporal cortex (CTX) and hippocampus (HPC) after addition of the various ASMs to slices perfused with 0 Mg^2+^/4-AP ± LPS. SVP, sodium valproate; PHE, phenytoin; PB, phenobarbital, MDZ, midazolam.

We tested the effect of specific ASMs that classically fail in FIRES patients, namely 800 μM sodium valproate, 50 μM phenytoin, 100 μM phenobarbital and 100 μM midazolam on 0 Mg^2+^ + 4-AP-evoked epileptiform activities in LPS-pre-exposed vs. naïve slices (Figure 4). Epileptiform events were quantified at T2 = 20–30 min after the beginning of drug perfusion (*protocol in* Supplementary Figure 1B). In slices exposed to 0 Mg^2+^ + 4-AP (Figure 4A), both phenytoin (*p* < 0.05), phenobarbital (*p* < 0.01) and midazolam (*p* < 0.05) partially reduced the frequency of FPs with residual epileptiform activity still detected, while sodium valproate was ineffective. In LPS-preincubated slices exposed to 0 Mg^2+^ + 4-AP (Figure 4B), only phenobarbital and midazolam partially reduced the frequency of FPs (*p* < 0.05; *p* < 0.01) while both phenytoin and sodium valproate were ineffective. The respective activity maps and raster plots are depicted in Figures 4D, E.

Since FP frequency analysis encompassed all epileptiform activities, we specifically analyzed *ictal events* and *SE events* (see Section 2.2 for definition) during 20–40 min perfusion. Notably, we found that while ASMs fully inhibited these activities in slices exposed to 0 Mg^2+^ + 4-AP (64% incidence in aCSF vs. 0% with ASMs), the same ASMs were only partially effective in LPS-pre-exposed slices (Figure 4C). In particular, ictal and SE events occurred in 86% of LPS-pre-exposed slices, in 57% of sodium valproate slices (4/7), in 33% of phenytoin and midazolam slices (2/6) and in 20% of phenobarbital slices (1/5 slices; Figure 4C).

These results indicate that seizure resistance to ASMs is exacerbated in LPS pre-treated slices.

### 3.4. Immunomodulatory drugs inhibit ASM-resistant epileptiform activity in lipopolysaccharide treated slices

**Figure 5:**
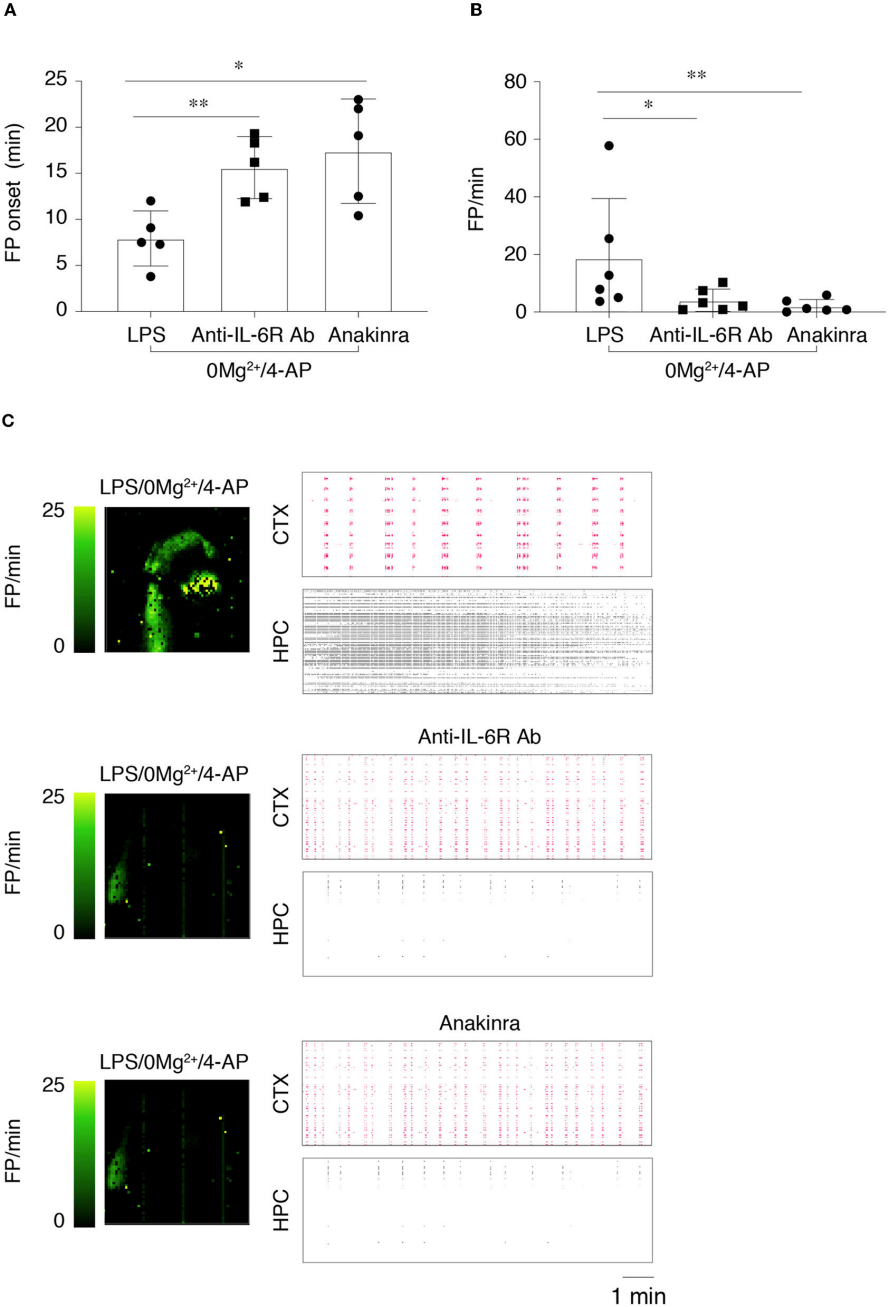
Effects of anti-IL-6R antibody and anakinra on epileptiform activity in lipopolysaccharide-treated slices. **(A, B)** The onset of the first field potential (FP) event **(A)** and FP frequency **(B)** in the various experimental groups (*n* = 6 slices/group). FP frequency was calculated *during T2* (20–30 min from the start of 0 Mg^2+^/4-AP perfusion). Slices were preincubated with aCSF containing LPS (10 μg/ml for 30 min, followed by 5 min aCSF washout), then perfused in aCSF ± anti-mouse IL-6R Ab (100 μM) or ± anakinra (1.3 μM) for 15 min followed by 0 Mg^2+^/4-AP ± drugs for 40 min. *p < 0.05; **p < 0.01 by Mann–Whitney test vs. respective control slices (aCSF, no added drugs). **(C)** Depicts representative activity maps (higher FP frequency corresponds to lighter green color) and raster plots in temporal cortex (CTX) and hippocampus (HPC) in slices perfused with 0 Mg^2+^/4-AP+LPS with or without the immunomodulatory drugs.

We investigated the effect of anakinra and the anti-IL-6R antibody on epileptiform activity in LPS-treated slices. To this aim, we modified the experimental protocol to take into account the time that the immunomodulatory drugs may require to counteract neuroinflammation. Thus, we perfused slices with aCSF containing the immunomodulatory drug for 15 min *prior* to switching to 0 Mg^2+^ + 4-AP perfusion solution. As for ASMs, the immunomodulatory drugs were perfused for further 40 min (Supplementary Figure 1C), and epileptiform events were measured during 20–30 min (T2). As depicted in Figures 5A, B, both immunomodulatory drugs showed similar effects by significantly delaying the time to onset of the first FP (*p* < 0.05; *p* < 0.01 vs. LPS/0 Mg^2+^ + 4-AP) and by reducing FP frequency (*p* < 0.05; *p* < 0.01 vs. LPS/0 Mg^2+^ + 4-AP). The respective activity maps and raster plots are depicted in Figure 5C. Importantly, ictal events and SE events in LPS/0 Mg^2+^ + 4-AP (83% incidence; Figure 4C) were abolished by both anakinra and the anti-IL-6R antibody (0% incidence: *depicted in* Figure 5C, HPC).

## 4. Discussion

We described a refined *in vitro* model of epileptiform activities induced in hippocampal/temporal cortex slices of naive mice by 0 Mg^2+^ + 4-AP (17). We choose this model since the evoked epileptiform events showed limited responsiveness to first and second line ASMs (17), as observed in FIRES patients (3–5). Moreover, the model used mouse hippocampal/temporal cortex slices, including subiculum, perirhinal and the entorhinal cortices. This circuitry is crucially involved in the epileptiform activity and neuropathology of FIRES, as shown by EEG and MRI studies. Finally, we used MEA recording for monitoring epileptiform events over the entire limbic circuitry.

To mimic salient clinical features of FIRES, we modified the original model (17) by taking into account the age of onset of FIRES and the cytokine storm. In particular, since the incidence of FIRES is higher in school-age children and young adults (although it may occur at any age), we used acute slices from 28 to 30-day old mice that approximates grade school age-puberty in humans (16). Moreover, the original model lacked the immune/inflammatory challenge which precedes seizure precipitation in FIRES. Thus, we preincubated slices for 30 min with the prototypical inflammatory agent LPS. This condition induced a prominent neuroinflammatory response in the slices that persisted after LPS washout for the entire time of electrophysiological recording. LPS-induced neuroinflammation included the induction of cytokines with both *in vitro* (20) and *in vivo* ictogenic properties, such as IL-1**β**, TNF (21, 22) and CXCL1 (23). In particular, the ratio of IL-1β to its receptor antagonist IL-1Ra was significantly increased by LPS vs. naïve slices, supporting a failure of endogenous antiinfammatory mechanisms to resolve neuroinflammation (13). A deficit in antiinflammatory mechanisms was described in epileptic foci of patients with ASM-resistant seizures (24), in the hippocampus of animal models of SE (13, 22) and patients with FIRES (25).

In accordance with the increased level of ictogenic cytokines/chemokines, LPS exacerbated the epileptiform activity evoked by 0 Mg^2+^ + 4-AP by increasing the frequency and burst duration of FPs, as well as the incidence of ictal/SE events. This evidence is in accordance with the increased frequency of evoked epileptiform discharges induced by LPS in immature rat hippocampal slices (18). Differently from our study, however, Gao et al. added LPS *after* epileptiform discharges occurred, and no drugs were tested. We provide, therefore, a new model for seizures in FIRES, where neuroinflammation contributes to the severity of epileptiform activity.

To determine the pharmacological responsiveness of seizures developing in the LPS-exposed slices, we tested the antiictogenic activity of specific ASMs that classically fail in FIRES patients, namely midazolam, phenytoin, phenobarbital and sodium valproate (4). Each ASM, used at its maximal therapeutic plasma concentration in humans, showed only a partial effect in reducing FPs in naïve slices exposed to 0 Mg^2+^ + 4-AP. Notably, in slices where neuroinflammation was induced by LPS, the refractoriness of epileptiform events to ASMs was exacerbated. In fact, phenytoin and sodium valproate were both ineffective on FP frequency, and midazolam and phenobarbital were only partially effective. Importantly, the incidence of ictal and SE events was increased in LPS-treated slices (86.6 vs. 64% without LPS). Moreover, these events were suppressed by ASMs in slices not pre-exposed to LPS, while they were only partially reduced by midazolam, phenobarbital and phenytoin, and unresponsive to sodium valproate, in LPS-exposed slices.

Next, we tested the effect of two immunomodulatory drugs, namely the anti-mouse IL-6R antibody (corresponding to tocilizumab in humans) and anakinra (recombinant human IL-1Ra) on epileptiform activity exacerbated by LPS. These drugs showed therapeutic effects on seizures and improved neurological outcomes in FIRES patients [*reviewed in* (3)]. Both immunomodulatory drugs at concentrations reflecting their maximal plasma or CSF therapeutic levels (26–28) drastically reduced epileptiform activities in LPS-treated slices, and abolished SE events. Thus, this refined *in vitro* model mimics both ASM-resistant seizures and their sensitivity to immunomodulatory drugs in FIRES. To maximize the rapidity of drug testing, we focused our analyses on the most active area (either hippocampus or temporal cortex, as shown by activity maps/raster plots) in the slice. Moreover, the epileptiform activity was quantified starting 20 min after perfusion of the ictogenic cocktail, when the epileptiform events were stably expressed and the drugs had time to act on their targets.

We propose this model as a first screening test to rapidly select potentially effective drugs for ASM-refractory seizures in FIRES patients. A limitation of the *in vitro* model is that it does not allow to control for drug penetration through the blood brain barrier and for PK/PD/toxicity issues which require to be addressed in an *in vivo* model. Recently, LPS-primed adult mice with increased hippocampal cytokine levels (e.g., IL-1β, TNF, IL-6) were shown to develop a more severe pilocarpine-induced SE compared to naïve mice. Similarly to our *in vitro* model, SE was refractory to various ASMs (29). Thus, this mouse model may represent a second step for *in vivo* validation after drug selection in the slice model.

Notably, the slice model reinforces the evidence that neuroinflammation in the limbic system exacerbates seizures and contributes to the mechanisms of ASM-resistance. Accordingly, in mouse model of SE refractory to benzodiazepines, the co-administration of anakinra and diazepam terminated SE (30). Drugs blocking the P2X7 receptors, which results in inflammasome inhibition, relieved SE resistance to various ASMs in mice (31). Our *in vitro* model, therefore, allows testing whether drug-resistance is relieved by combining antiinflammatory drugs with ASMs. Understanding whether neuroinflammation is a factor involved in seizure severity and refractoriness to ASMs would prompt early addition of anti-inflammatory drugs to the conventional treatment protocols in FIRES patients.

In conclusion, the *in vitro* experimental data support that cytokine pathways, mediated for example by IL-1β, TNF and CXCL1/IL-8, are involved in ASM-resistant seizures in FIRES. These factors can be targeted by drugs with immunomodulatory properties, such as anakinra and tocilizumab, or by new investigational drugs against other inflammatory targets that are emerging in the preclinical literature (32, 33). Since the etiopathogenesis of FIRES is still unknown, this model mimics the pharmacological response of seizures to clinically used drugs in FIRES patients. The model, therefore, could facilitate drug screening before *in vivo* testing, allowing a faster path to the clinical use of new effective treatments for FIRES that are urgently needed.

## Data availability statement

The raw data supporting the conclusions of this article will be made available by the authors, without undue reservation.

## Ethics statement

The animal study was reviewed and approved by Mario Negri Institute Animal Care and Use Committee, and by the Italian Ministry of Health.

## Author contributions

MC and MDN conducted the experiments and analyzed the data. IC conducted RT-PCR experiments and analyzed the data. AV supervised the study and together with MC designed the experiments and wrote the manuscript. All authors have read and approved the final version of the manuscript.
