# Supplementary material for: An in vitro model of drug-resistant seizures for selecting clinically effective antiseizure medications in Febrile Infection-Related Epilepsy Syndrome
Source: Front Neurol. 2023 Mar 22;14:1129138. doi: 10.3389/fneur.2023.1129138 (PMC10074483; doi:10.3389/fneur.2023.1129138)
Supplement: Supplementary file 1 [file Data_Sheet_1.pdf]

Supplementary Material

An in vitro model of drug-resistant seizures for selecting clinically effective antiseizure medications in Febrile Infection-Related Epilepsy Syndrome (FIRES)

Milica Cerovic*, Martina Di Nunzio, Ilaria Craparotta, Annamaria Vezzani *

* Correspondence: Corresponding Author: annamaria.vezzani@marionegri.it
milica.cerovic@marionegri.it

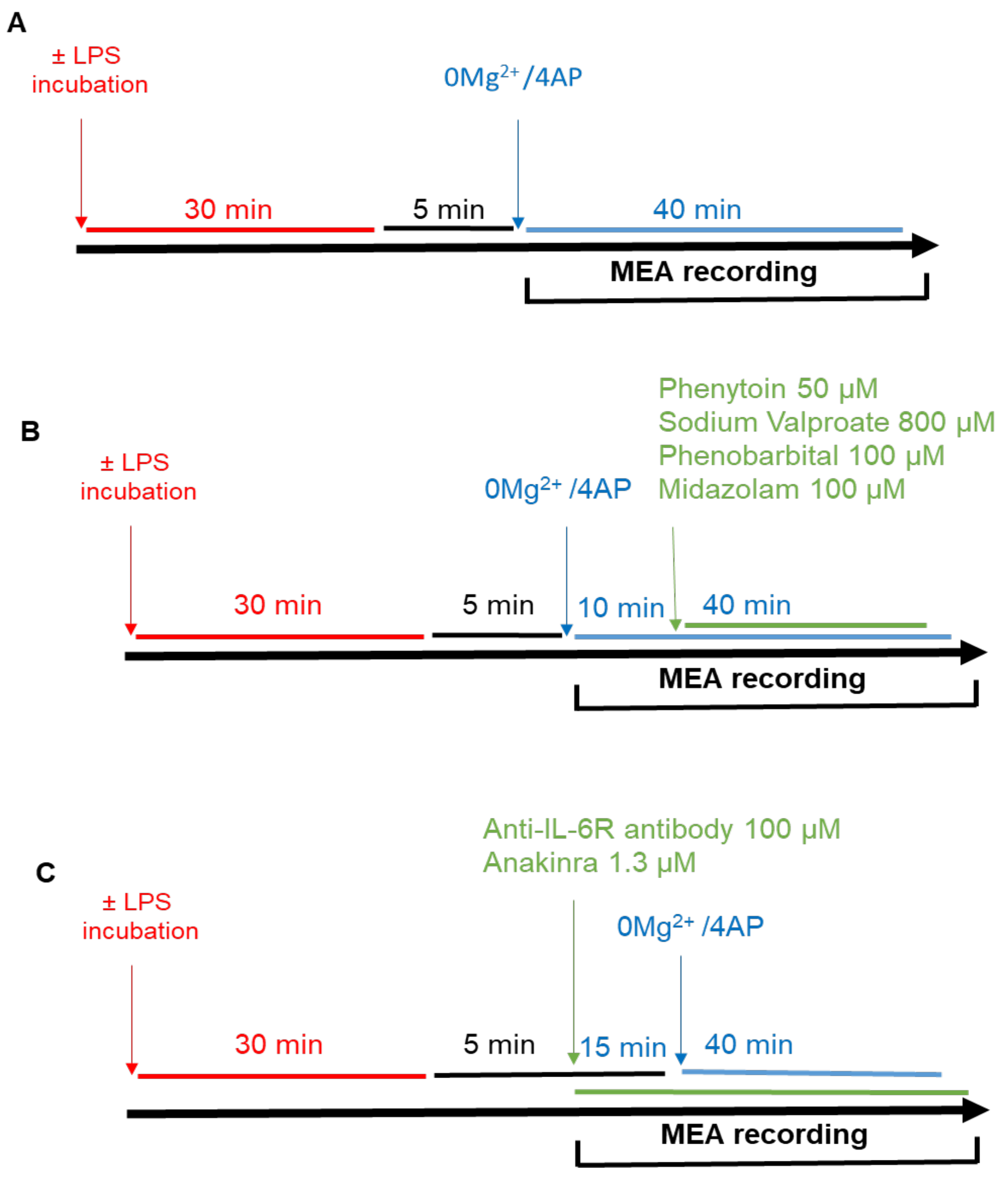

Supplementary Figure 1. A: Experimental protocol of LPS challenge. After 30 min incubation with 10 µg/ml LPS, slices were transferred to recording MEA chip and washed-out for 5 min. Then, slices were perfused with $0Mg^{2+}$+100 µM 4-AP solution for 40 min to evoke synchronized field potentials (FPs). Control slices were incubated in aCSF and similarly handled.

B: Experimental protocol of ASM treatment. After incubation with LPS for 30 min, slices were transferred to the MEA chip and washed-out for 5 min with aCSF. Then, slices were perfused with $0Mg^{2+}$+100 µM 4-AP solution and epileptiform events were recorded for at least 10 min after the occurrence of the first FP. ASM was added in the $0Mg^{2+}$+100 µM 4-AP solution at the selected concentration, and epileptiform activity was recorded for additional 40 min.

C: Experimental protocol of immunomodulatory drug treatment. After incubation with LPS for 30 min, slices were transferred to recording MEA chip and washed-out for 5 min with aCSF, then slices were perfused for 15 min with aCSF ± immunomodulatory drug at the selected concentration. Subsequently, slices were perfused with $0Mg2^{+}$+100 µM 4-AP solution ± immunomodulatory drug and epileptiform events were recorded for 40 min.
